# Photo-Crosslinked Polymeric Matrix with Antimicrobial Functions for Excisional Wound Healing in Mice

**DOI:** 10.3390/nano8100791

**Published:** 2018-10-05

**Authors:** Ming-Hsiang Chang, Yu-Ping Hsiao, Chia-Yen Hsu, Ping-Shan Lai

**Affiliations:** 1 Department of Chemistry, National Chung Hsing University, Taichung 402, Taiwan; sistericy@hotmail.com (M.-H.C.); clhsqoo@gmail.com (C.-Y.H.); 2 Institute of Medicine, School of Medicine, Chung Shan Medical University, Taichung 402, Taiwan; missyuping@gmail.com; 3 Department of Dermatology, Chung Shan Medical University Hospital, Taichung 402, Taiwan

**Keywords:** photo-crosslinked film, antibacterial effects, wound dressing

## Abstract

Wound infection extends the duration of wound healing and also causes systemic infections such as sepsis, and, in severe cases, may lead to death. Early prevention of wound infection and its appropriate treatment are important. A photoreactive modified gelatin (GE-BTHE) was synthesized by gelatin and a conjugate formed from the 3,3′,4,4′-benzophenone tetracarboxylic dianhydride (BTDA) and the 2-hydroxyethyl methacrylate (HEMA). Herein, we investigated the photocurable polymer solution (GE-BTHE mixture) containing GE-BTHE, poly(ethylene glycol) diacrylate (PEGDA), chitosan, and methylene blue (MB), with antimicrobial functions and photodynamic antimicrobial chemotherapy for wound dressing. This photocurable polymer solution was found to have fast film-forming property attributed to the photochemical reaction between GE-BTHE and PEGDA, as well as the antibacterial activity in vitro attributed to the ingredients of chitosan and MB. Our in vivo results also demonstrated that untreated wounds after 3 days had the same scab level as the GE-BTHE mixture-treated wounds after 20 s of irradiation, which indicates that the irradiated GE-BTHE mixture can be quickly transferred into artificial scabs to protect wounds from an infection that can serve as a convenient excisional wound dressing with antibacterial efficacy. Therefore, it has the potential to treat nonhealing wounds, deep burns, diabetic ulcers and a variety of mucosal wounds.

## 1. Introduction

Wound healing is a complex process which includes five distinct phases: hemostasis, inflammation, migration, proliferation, and remodeling [1]. Most often, healing arrest happens when wounds are infected in the inflammatory phase [2]. During wound therapy, bacterial infection and wound sepsis are the major clinical problems which lead to mortality and morbidity in patients [3,4]. To mitigate these problems, antimicrobial agents can be used through an adsorption of bacteria that destroys the normal function of bacterial proteins or damages the integrity of cell membranes and, therefore, may lead to bacterial growth inhibition and cell death [5,6,7,8]. Among these antimicrobial agents, silver-related compounds, such as silver nitrate and silver sulfadiazine for antibacterial therapy has been demonstrated and, also, its adverse effects, like cell toxicity, and gray and black discolorations, are reported [9,10,11]. Recently, silver nanoparticles with greater mobility and transdermal penetration were reported to have remarkable antibacterial abilities [12,13,14]. Unfortunately, the silver nanoparticles can induce cellular apoptosis via reactive oxygen species (ROS)- and c-jun N-terminal kinase (JNK)-dependent mechanisms, hence posing another problem of nanotoxicity [15].

A smart and reasonable polymeric matrix wound dressing is also an alternative strategy to overcome the bacterial pathogen-induced wound infection. The cationic polymers, which mimic the function of cationic antimicrobial peptides through bacterial membrane disruption, were synthesized by formation of a low degree polymerization of cationic polymers with lipophilic tails within twelve carbon lengths [16,17]. A suitable degree of styrene sulfonate monomer incorporated in heparin-mimicking polymer not only promoted fibroblast growth factor-2 (FGF2) binding to its receptor, but also increased the cell proliferation in vitro, enhancing the rate of wound healing [18]. Introducing the antioxidant agents, such as curcumin and astaxanthin, by either blending or grafting into polymer, reduce the bacterial growth, as well as increased the wound-healing rate, as compared to its bare polymer [19,20]. Moreover, wound dressing-based polymers with good tensile, elasticity, and shape recovery characteristics, such as poly(siloxane-urethane) elastomers with self-remodeling capability via Diels-Alder (DA) and retro-DA reaction, provided not only the feasibility to blend antioxidants or antibacterial agents into polymers but, also, preserve the dressing integrity on wounds at the joint, where the curvature changes frequently [21].

Natural heteropolysaccharide chitosan, made from deacetylated chitin, acts as a good biomaterial with biodegradable and great biocompatibility properties for medical and antibacterial applications [22,23]. Chitosan, with its positive charge, demonstrates an antibacterial mechanism that can interfere with the cell surface via electrostatic interaction, disturb the permeability of cell walls and, therefore, cause leakage from the cytosol and subsequent cell death [24,25]. In addition to this, the internalized chitosan can form a stable complex with deoxyribonucleic acid (DNA) and alter the chromosome structure, thereby inhibiting ribonucleic acid (RNA) biosynthesis and reducing cell viability [26]. However, the antibacterial mechanism of chitosan may depend on its molecular weight. A high molecular weight of chitosan results in a better antibacterial activity against Gram-positive bacteria that may be due to the ability of chitosan to accumulate outside the bacterial membrane to form a compact membrane that prevents nutrients reaching from the bacterial cell [27,28]. On the other hand, for Gram-negative bacteria, chitosan of lower molecular weight (less than 5000 g/mol) easily penetrates into the structure of the cell wall to interfere with the cell metabolism due to cell apoptosis [29].

**Scheme 1:**
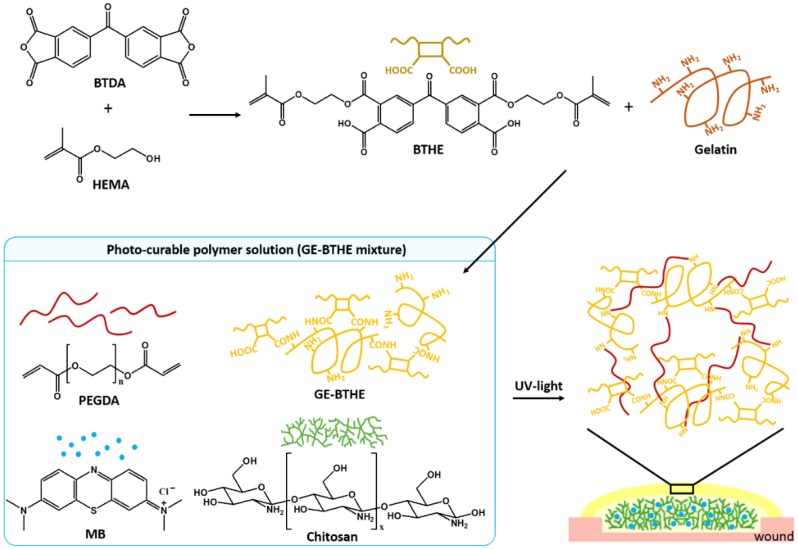
Representative synthesis procedure of GE-BTHE and the procedure of GE-BTHE mixture forming wound dressing post UV light-irradiation.

More recently, another strategy based on photodynamic therapy (PDT), named photodynamic antimicrobial chemotherapy (PACT), was investigated for topical antimicrobial activity [30,31]. PDT is a photochemical process which is used to generate localized cell death, and involves the activation of a photosensitizing drug in the target area with light in the presence of molecular oxygen [32]. Our group has reported several PDT-related studies for cancer therapy, including the photo-triggered drug/gene release and nanoparticle-based photosensitizer delivery systems [33,34,35,36,37]. PDT is not only a therapy for cancer and other diseases but is also an antimicrobial strategy [38,39]. For wound therapy, PACT has been demonstrated successfully to kill various microbes and rapidly control topical wound infection in vivo [40,41]. Thus, in this study, PACT was used in combination with chitosan in topical wounds, to see whether this approach would reveal any synergistic effects useful for antibacterial wound dressing. In addition to this, a rapid wound dressing, which can provide a moist environment leading to rapid granulation and re-epithelialization is also required [42]. GE-BTHE (BTDA/HEMA modified liquid-phase gelatin), a light crosslinkable polymer containing two acrylic vinyl groups (from HEMA) and two carboxylic groups (from BTDA) bonding with amino groups of gelatin, was revealed to be fast-film-forming with poly(ethylene glycol) diacrylate (PEGDA) after ultraviolet (UV) irradiation (Scheme 1) [43]. PEGDA is a low toxicity poly(ethylene glycol)-derivate and acts as a crosslinker to connect with the conjugated GE-BTHEs via the reaction between acryloyl groups on PEGDA and amine groups on gelatin under UV light-irradiation [44]. Moreover, the tensile strength of this UV-cured membrane was positively correlated to its degree of swelling, and the strength, as well as curing time, could be tuned according to the molecular weight of PEGDA used, suggesting that GE-BTHE mixtures applied on wound dressings could be potentially feasible. As compared with the smart polymeric matrix wound dressing, the antibacterial effect in this study was concentration-dependent on chitosan and MB, and not structure-dependent on the synthetic polymers, making it easier and feasible as a wound dressing. Herein, we prepared a chitosan/photosensitizer GE-BTHE photo-crosslinked polymeric matrix as a rapid wound-dressing material with two types of antimicrobial strategies for wound healing. Therefore, the UV and FT-IR spectra of the ingredients in GE-BTHE mixture were measured. The composition of GE-BTHE mixture for in vivo antibacterial efficiency was according to the result of in vitro antibacterial activity using a colony assay. The overall in vivo antibacterial efficacy was evaluated on the *Staphylococcus aureus* 71 (*S. aureus*)-infected full skin thickness excisional wounds as compared with bare-wounds.

## 2. Experiment 

### 2.1. Chemicals and Instruments

Gelatin (food grade, type A, 225 Bloom, M_w_ = 50,000), poly(ethylene glycol) diacrylate (PEGDA average M_n_ = 700), chitosan (75–85% deacetylated, M_w_ = 100,000), and methylene blue (MB; as photosensitizer) were obtained from Sigma-Aldrich (St. Louis, MO, USA), 2-hydroxyethyl methacrylate (HEMA 96%, *N*,*N*′-dicyclohexyl-carbodiimide (DCC 99%), 4-dimethylamino-pyridine (DMAP 99%), 3,3′,4,4′-benzophenone tetracarboxylic dianhydride (BTDA 98.5%, M_w_ = 322.23), methanol (99.5%), and acetone (98%) were purchased from ACROS Organics (Fair Lawn, NJ, USA). DPBS and trypsin were purchased from Invitrogen^©^ (Carlsbad, CA, USA). All chemicals were reagent grade and used without further purification, unless otherwise noted. GE-BTHE was synthesized according to previous report [43]. Ultraviolet-visible (UV-vis) spectra of prepared samples were recorded on Hitachi Model U-3010 spectrophotometer (Tokyo, Japan) and Fourier transform infrared spectra (FT-IR) were obtained on a Bruker Tensor 27 spectrophotometer (Billerica, MA, USA). The fluorescence spectrum was analyzed by using a Jasco FP-6300 spectrofluorometer (Tokyo, Japan). UV-curing was performed using a UV light (~300 nm) and a laser to irradiate samples.

### 2.2. In Vitro Colony Assay

To evaluate the antibacterial activity of each material, *Escherichia coli* DH5α (*E. coli*; Gram-negative bacteria; ~10^8^ cells/mL) and *Staphylococcus aureus* 71 (*S. aureus*; Gram-positive bacteria; ~10^9^ cells/mL) were incubated with various concentration of chitosan, GE-BTHE, or MB, and all procedures were carried out in subdued light. MB groups for the evaluation of PDT were divided into irradiated and non-irradiated groups. In the irradiated groups, cultured cells were exposed to light irradiation (660 nm, 19.5 mW) for 15 min to stimulate the photochemical reaction. After that, cells in all groups were diluted serially, transferred to nutrient agar plates, and then cultured at 37 °C for 24 h for cfu (colony-forming unit) observation. Experiments were carried out in triplicate.

### 2.3. Prepartation of Antibacterial and Photocurable Polymer Solutions

The photocurable polymer solution was prepared as a mixture of GE-BTHE/chi- tosan/MB/PEGDA. Samples of various GE-BTHE, chitosan, MB ratios were prepared for antibacterial studies. For the bacteriostasis in vitro test, the best ratio based on the optimum antibacterial results of GE-BTHE, chitosan, and MB were found 48.19 wt %; 51.63 wt %; and 0.18 wt % respectively. For the in vivo study, the photocurable polymer solution for wound healing was prepared with a ratio of 1.46 wt %; 1.56 wt %; 0.0056 wt %; 92 wt %; and 4.48 wt % of GE-BTHE, chitosan, MB, PEGDA, and PBS, respectively. After mixing in an Eppendorf tube with sonication (until homogeneous), samples were stored at 4 °C.

### 2.4. Bacteriostasis In Vitro

To examine an antimicrobial effect of MB-mediated PDT, inhibition zone analysis was performed, and broth culture was inoculated with *E. coli* (Gram-negative bacteria) and *S. aureus* (Gram-positive bacteria). According to the disc agar diffusion (DAD) method, photocurable polymer solution was pipetted with 40 μL onto a paper disc (8 mm in diameter), then attached to the inoculated culture and cultured for 24 h in an incubator at 37 °C. The efficiency of bacteriostasis of the mixed polymer solution in vitro was assessed by measuring the diameter of the inhibition zone with a caliper. The larger the diameter observed, the stronger the bacteriostasis. Samples of various GE-BTHE, chitosan, MB ratios were prepared for different inhibition zones.

### 2.5. Wound Dressing In Vivo

All animal care and experimental protocols were approved by the Institutional Animal Care and Use Committee (IACUC) of National Chung Hsing University (IACUC Approval Number 106-045). Mice weighing 25 g (female, 8–12-weeks-old) were used in this study. After being anesthetized with Balanzine^®^, three round wounds (about 25 mm^2^) were prepared on the dorsum of each mouse using a sharp pair of scissors and tweezers. The photocurable polymer solution (20 μL) was placed onto each wound as wound-dressing glue, and then followed 20 s of UV irradiation for in situ film-forming. Wounds were monitored by photo records on day 3, 6, 9, and 13 during the recovery time. After 21 days, the wounds were healed, and 5 × 5 mm^2^ of the skin, including the wounds, were removed from each mouse for histological examination.

### 2.6. Histological Analysis

For histological analysis, the wounded tissues were dissected at day 21 after cervical dislocation. Isometric continuum cut sections were obtained using a microtome in transverse and vertical planes from the skin of each wound. The tissues were then fixed in formalin and embedded in paraffin. The paraffin-embedded 3 μm sections were analyzed by hematoxylin and eosin (H&E) staining. Hematoxylin stains cell nuclei blue, whereas eosin stains cytoplasm, connective tissue, and other extracellular substances pink or red. Images of the stained sections were acquired using a light microscope (BX 50, Olympus, Tokyo, Japan) equipped with a digital camera (DP 20, Olympus, Tokyo, Japan). Wound area, distribution of subcutaneous cells (such as neutrophil, fibroblast, and lymphocyte, etc.) and number of hair follicles were included for the wound score evaluation. The former two criteria are the evaluated points for early stage wound healing. In the late stage (after 21 days) the number of hair follicles may help to identify the condition of wound recovery. Quantitative analysis of wound healing, such as skin healing of epithelial cell regeneration (re-epithelialization), granulomas (granulation), and angiogenesis levels, was according to the methods from Altavilla et al. and Shacklford et al. [45,46].

## 3. Results and Discussion

### 3.1. Antibacterial Effects of Each Reagent

The antibacterial effects of each material in the polymeric matrix, including GE-BTHE, chitosan, PEGDA (crosslinker), or MB (photosensitizer), with or without light irradiation, were evaluated against *E. coli* and *S. aureus* with various concentrations. As shown in Table 1, a chitosan concentration at 3300 μg/mL has a significant inhibitory effect. GE-BTHE exhibited low bacterial inhibition at the concentration of 1600 µg/mL. However, it was slightly toxic towards both *E. coli* and *S. aureus* when the concentration was increased to 4000 µg/mL. MB exhibited no inhibitory effect without red light-irradiation; however, after red light-irradiation, it showed significant antibacterial activity to *E. coli* at 8 μg/mL concentration. However, in our results, MB showed less antibacterial activity towards *S. aureus* for all concentrations. In general, chitosan (0.1% w/v) exhibits a stronger antibacterial effect towards Gram-positive bacteria than Gram-negative bacteria [27]. It has been reported that chitosan has a higher antibacterial activity, which markedly inhibits the growth of most bacteria tested. The inhibitory effects differ with the molecular weight of chitosan and the particular bacterium. As shown in Table 1, chitosan exhibited a better ability to inhibit the growth of *S. aureus* than *E. coli*, which indicates that Gram-positive bacteria were inhibited more efficiently by chitosan at the same concentration of Gram-negative bacteria. In addition, MB-mediated PDT exhibits both the efficient antitumor efficacy and also photo-inactivated activities towards various microorganisms and viruses via ROS generation [47,48,49,50]. Biel et al. explained that MB exerts a bactericidal efficacy at a concentration of 180–250 μM, reducing the cfu by a maximum number of 2.512 × 10^8^ for *E. coli* [48]. The results of this study showed efficient inhibition towards *E. coli* using GE-BTHE and MB with light (800 μg/mL, 8.3 × 10^7^ cfu reduction in GE-BTHE and 80 ng/mL, 2.4 × 10^8^ cfu reduction plus light in Table 1). In fact, the bactericidal efficacy is due to the different gene sequence of *E. coli* between Biel’s and our group, so the bactericidal efficacy may not be compared. Moreover, the viabilities of *E. coli* and *S. aureus* were further reduced, respectively, around 80.6% and 15.2% upon light triggering MB to generate PACT activity at 8 μg/mL of MB, as compared to the MB-treated bacteria without UV light-irradiation. These results indicated that both *E. coli* and *S. aureus* were sensitive to the antibacterial activity of chitosan at a concentration of 3300 μg/mL, but the PACT effect of MB was superior in *E. coli* than in *S. aureus* at the concentration higher than 4 μg/mL. Although better PACT effects on *E. coli* were exhibited, we challenged the excisional wounds with *S. aureus* prior to the photocurable polymer solution treatment to evaluate the effect of forming wound dressing on long-term wound healing. Therefore, the concentrations of each component of the glue, for in vivo wound dressing with the highest antimicrobial efficacy, were chosen as follows: chitosan, 3300 μg/mL; MB, 8 μg/mL; and GE-BTHE, 1600 μg/mL.

### 3.2. Characterization of the Photocurable Polymer Solution

**Figure 1:**
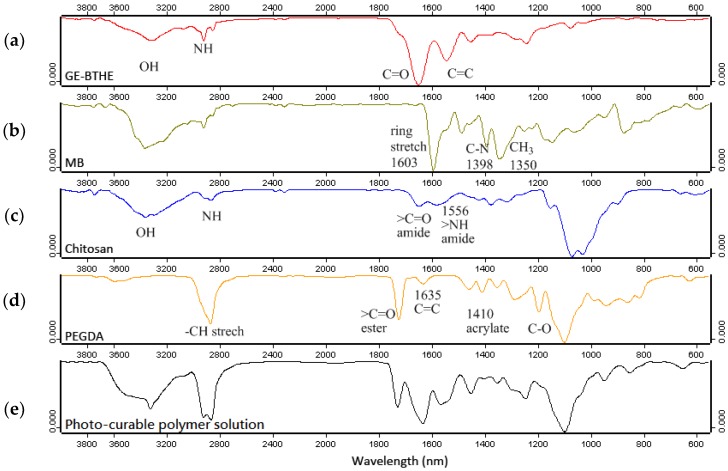
FT-IR spectra of (**a**) GE-BTHE; (**b**) MB; (**c**) chitosan; (**d**) PEGDA; and (**e**) the mixture of photocurable polymer solution (including the above components).

According to the effective concentration of each component as described above, the matrix-based photocurable polymer solution was prepared as antibacterial wound-dressing material. Figure 1 shows the ATR-FTIR spectra for analysis of the structural formula of the five materials (GE-BTHE, MB, chitosan, PEGDA, and the mixture of photocurable polymer solution). Figure 1a indicates the unirradiated GE-BTHE, showing –OH, –NH, and C=O group absorptions at 3300, 2900, and 1680 cm^−1^, respectively. The C=C bond stretching appears at 1480 and 1550 cm^−1^. Figure 1b shows the IR spectra of MB, which exhibits a ring stretch at 1603 cm^−1^, symmetric stretch of C–N at 1398 cm^−1^, and symmetric deformation of –CH_3_ at 1354 cm^−1^. Figure 1c shows the IR absorptions of the –OH and –NH group of chitosan at 3360 cm^−1^ and 2890 cm^−1^, respectively. The absorption of the amide C=O bond shows at 1650 cm^−1^ and –NH bending at 1556 cm^−1^. The characteristic peaks for the acrylate groups of PEGDA (i.e., those at about 812 cm^−1^, 1190 cm^−1^, and 1410 cm^−1^) shows in Figure 1d. The bottom panel Figure 1e shows the ATR-IR spectra of all components, GE-BTHE, MB, chitosan, and PEGDA. The C=C stretching of GE-BTHE at 1480 and 1550 cm^−1^ disappeared owing to crosslinking with PEGDA. The acrylate group (1410 cm^−1^) of PEGDA also disappeared in the ATR-IR spectra of the mixture of photocurable polymer solution. The specific absorptions of MB (1603 cm^−1^ and 1354 cm^−1^) and chitosan (1000–1100 cm^−1^) were not clearly observed because the crosslinked structure covered MB and chitosan, and both specific absorptions were shielded.

**Figure 2:**
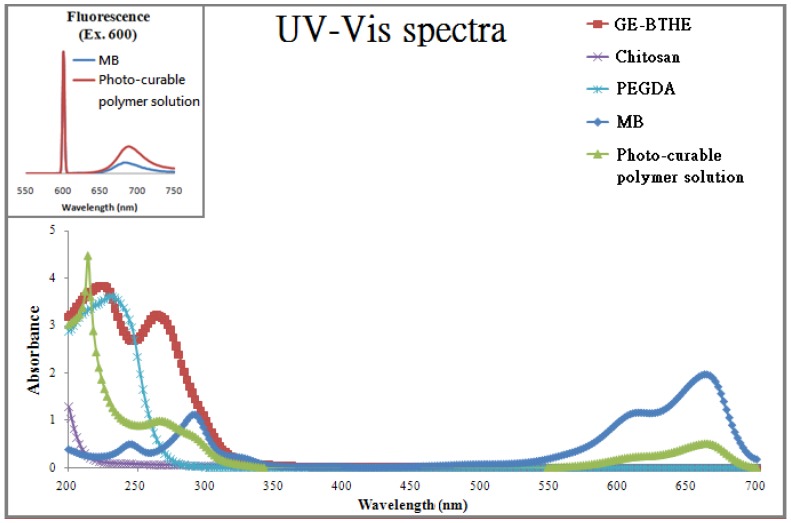
UV-vis spectra of GE-BTHE, MB, chitosan, PEGDA, and the photocurable polymer solution. The upper-left inset shows the fluorescence of MB and photocurable polymer solution.

Figure 2 shows the representative UV-vis spectra of each component and the mixture of photocurable polymer solution. Obviously, chitosan and PEGDA has no significant absorptions in the visible region (400–700 nm). GE-BTHE has a strong absorption at 267 nm, whereas MB has multiple absorptions at 290, 610, and 665 nm. According to the Woodward’s rules, the benzoyl derivatives have a basic value of UV absorption at 230 nm. The real value of absorption depends on the substitution type of electron-releasing or -withdrawing. The absorption at 235 nm comes from the benzophenone structure of GE-BTHE, and 245 nm comes from the anthracene-like aromatic structure of methylene blue, which makes the absorption shift to the right. Therefore, the absorption located at 260–300 nm on the green line can be used to activate C=O of GE-BTHE and produce free radicals, which is used to carry out photocuring between GE-BTHE and PEGDA via vinyl groups on both the compounds. Otherwise, the inset shows the fluorescence spectra of photocurable polymer solution and MB with the excited wavelength at 600 nm. The curves of photocurable polymer solution and MB have closely emitted wavelengths, but photocurable polymer solution has a 6 nm “red shift” compared to MB, due to aromatic conjugation.

### 3.3. Surface Morphology

**Figure 3:**
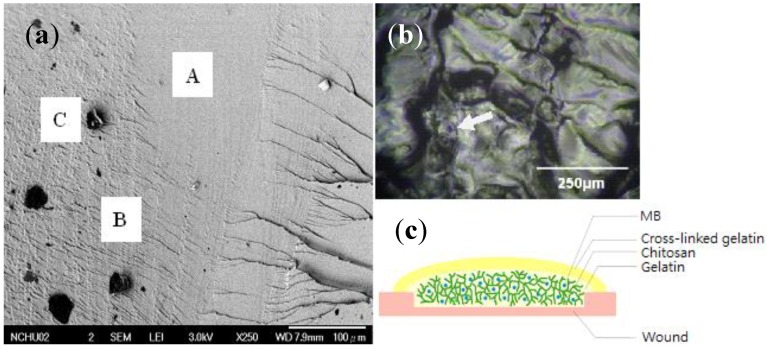
(**a**) SEM images of three different textures: (A) gelatin; (B) chitosan; and (C) MB; (**b**) Matrix observed by optical microscope (40×), arrow indicates a MB particle; (**c**) The supposed scheme of the matrix for wound healing and antimicrobial action.

Figure 3a shows the SEM image of the matrix with three main components resulted from gelatin, chitosan, and MB: (A) exhibited good film-forming, and the crosslinking of gelatin resulted in a flatter surface; (B) the dendritic texture of chitosan is due to its bad film-forming property; and (C) the better conductivity and resonance of MB caused some aggregation. The formation of the dark particles is because of the electron beam bumping under the electron microscope. The freeze-drying process of preparing the SEM samples caused large branches on the right-hand side of the SEM image. Figure 3b shows the matrix observed by optical microscope (40×) and the arrow indicates the MB particle. Figure 3c indicates the tentative scheme of the matrix on the wound for healing and antimicrobial activity. According to our in vitro results, the toxicity testing of gelatin shows the bacteria will be killed when the solution has contents of GE-BTHE higher than 4000 μg/mL, and this may be caused by ethanol (co-solvent). Of course, the toxicity may come from the unreacted functional groups, such as acrylate group. If gelatin is crosslinked as a dressing, it will be closer to the surface, the location of the nearest light source, then chitosan and MB will be pressed under the gelatin layer. In addition, MB has been sonicated into smaller molecules when mixed, and it served as a photosensitizer to induce the PDT effect, and an antibacterial effect of chitosan was also exerted. MB-mediated PDT (MB-PDT) has been applied in the sterilization of blood products owing to a number of advantages, including its high safety profile, low cost, minimal leftovers, and low reduction of coagulation factors [51]. Thus, when irradiation is performed with a specific light source, this matrix acts as an antimicrobial agent. The PDT effect of MB first kills bacteria, then chitosan acts as an antibacterial agent and also assists wound recovery after gelatin crosslinking.

### 3.4. Bacteriostasis In Vitro: Disc Agar Diffusion Method (DAD)

There are two bacteria (including *E. coli* and *S. aureus*) in the bacteriostatic diameter results (shown in Table 2) which indicate the relationship between the antibacterial effect and the GE-BTHE/chitosan ratio. Park et al. mentioned that PDT has only a minor antimicrobial effect on *E. coli* with chlorin e6 in the inhibition zone [52]. Caminos et al. also mentioned that PDT only can work on the suspension bacteria, because the photosensitizer cannot completely conjugate the membrane of bacteria if the bacteria are cultured on agar plates [53]. Our pre-testing showed that irradiating on methylene blue with a concentration higher than 4 µg/mL only caused the effect of killing bacteria on *E. coli* (no effect on *S. aureus*), but did not alter the bacteriostatic diameter using the DAD method. If there is no irradiation, MB does not have a PDT function for the killing of bacteria, so the changes in the concentration of MB did not influence the effect of bacteriostasis. The reason is probably that the DAD method relies on the antibodies releasing from the disc to inhibit the bacteria. Therefore, the bacteriostatic diameter experiment was conducted without irradiation. The bacteriostatic diameter increased with increasing amounts of chitosan, and GE-BTHE also had a mild effect, so increasing the GE-BTHE/chitosan ratio caused a slight decrease in the bacteriostatic diameter. Both *E. coli* and *S. aureus* had the similar results in bacteriostasis.

Our disc agar diffusion results differed from those of a previous study [54]. If our samples follow their gelatin/chitosan ratio, the theoretical result should not show the bacteriostatic effect in Gram-negative bacteria. However, our results show an inhibition zone at the ratio of 5/3 (close the paper’s result of 1.6) with the bacteriostatic diameter 10 mm. A possible reason for this result is the different species of bacteria: their test bacteria were Gram-positive and ours were Gram-negative. Furthermore, the molecular weight of chitosan used in the previous study was 5000 Da, and some studies have shown that a low molecular weight of chitosan exerts a lesser inhibition effect on Gram-positive bacteria [55,56,57]. The explanation for this is that low-molecular-weight chitosan cannot attach to the surface of Gram-positive bacteria wall, so some nutrition has the chance to enter the membrane of the bacteria and keep the bacteria alive. On the contrary, Gram-negative bacteria have a thinner membrane, and chitosan of low molecular weight can easily enter and disrupt the bacteria. Therefore, for better inhibition, the GE-BTHE/chitosan ratio should be lower.

In addition to this, the literature report also shows that Ag nanoparticles (nAg) have good antibacterial effects, such as an antimicrobial dressing [58]. Crosslinked nAg-loaded gelatin hydrogel pads were observed by the agar diffusion method to exhibit bacteriostasis (*E. coli*), with an inhibition zone of 2.5 mm on average (measured from the edge of the sample to the edge of the clear zone). Comparing this result with our findings (average up to 3 mm), both our compound and the nAg-loaded derivatives (average up to 2.5 mm) have good antibacterial effects.

### 3.5. Wound Dressing In Vivo

**Figure 4:**
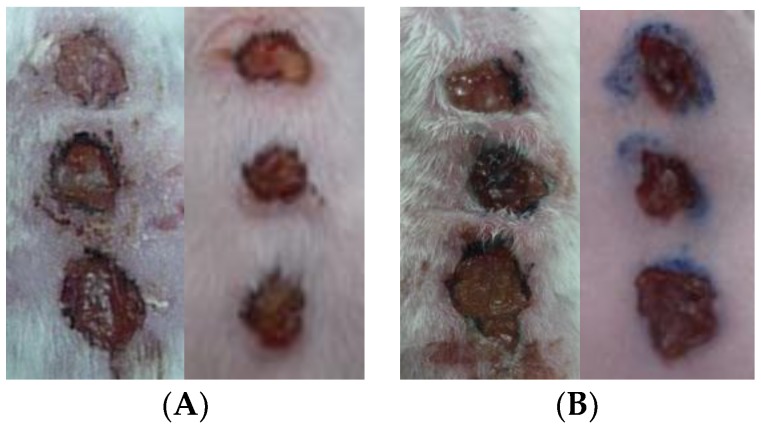
Comparisons of wound self-recovery and wound dressing in vivo. (**A**) 3 days after wound infliction; (**B**) after 20 s following UV-curing 1 day post-wounding. (Left: *E. coli*; right: *S. aureus*).

After injury, inflammation of the skin will occur as a red, swollen, hot phenomenon that usually lasts for three to four days but, in the elderly people, will remain for a week. Subsequently, granulation tissue begins to grow, epithelial cells proliferate and migrate to cover the granulation tissue, collagen synthesis begins, new blood vessels grow, and the wound begins to shrink, often to lasting ten days from the proliferative phase weeks. Finally, the wound will scar, and becomes covered with narrow self-collagen fibers arranged in a regular manner; unnecessary new blood vessels begin to degenerate and atrophy, maturity usually being reached between two to six weeks but, for complete healing of the wound, a two-year wait is required [59,60]. If the wound is not healing, it is easy to contract invasive infections caused by bacteria, which leads to complications, potentially life-threatening. Figure 4 shows a comparison of untreated wounds after 3 days and photocurable polymer solution-treated wounds after 20 s of irradiation. The irradiated GE-BTHE mixture can be transferred quickly into artificial scabs to protect wounds from infection, whereas untreated wounds form a scab naturally after about 3–7 days. Trauma can stop abundant bleeding, then form an important scab. Although, we did not have any cases of heavy bleeding in this study, but the scab formed sooner and the wound could heal itself as well. The most important thing is to stop bleeding by quickly transforming a wound-dressing glue into a scab. Moreover, the safety issue of the photocurable polymer solution might be minimal in inducing hemolysis. Similar ingredients utilized in this study, formed hydrogel from poly(HEMA-itaconic acid-PEGDA) triblock copolymer, showed less than 1% of hemolysis [61]. Furthermore, the gelatin and PEGDA components in the film-forming wound dressing were speculated to impair the mechanical strength of dressing by time, due to the intrinsic biodegradable characteristic of themselves [62,63]. The molecular weight of PEGDA in the wound dressing also influences the mechanical property of dressing that could be tuned to apply on a different wound area and position.

For wound healing, the main substrates were mostly collected by chitosan and gelatin for both the artificial skin and wound dressing [64]. When integrating dressing functionalities, the material needs to be modified so that it has a better antibacterial property or a faster healing effect [65]. Studies comparing the degree of wound healing with chitosan–nanocrystalline silver dressings showed superior healing rates (~89%) to those obtained with silver sulfadiazine dressings (~68%) and chitosan film (~74%) [9]. The main purpose of adding nanocrystalline silver is the use of silver nanoparticles to reduce toxicity and achieve a better antibacterial effect. The purpose of adding MB in our experiment is to kill bacteria and reduce inflammation, the rapid bactericidal effect assisting to achieve rapid wound healing. In addition, some studies state that chitosan with gelatin, hyaluronic acid, and TiO_2_ nanoparticles can be used as biodegradable artificial skin [66]. After 12 days, wounds showed an obvious healing phenomenon, and the manufactured wound film fell off at this time. We also found that the scabs fell off more slowly, the reason for this probably being differences in water absorption and chitosan–gelatin co-blends [67].

### 3.6. Wound-Healing Evaluation

**Figure 5:**
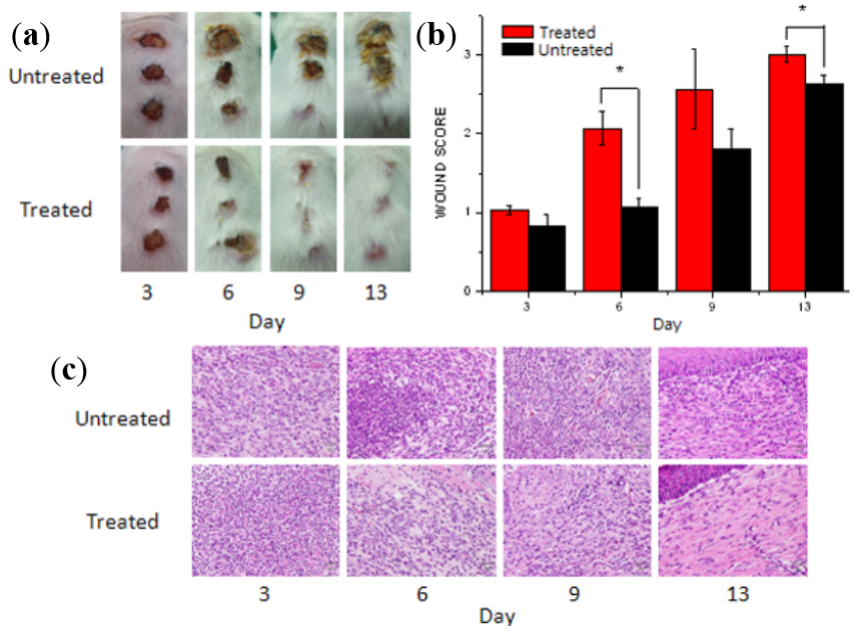
Effects of mixture of photocurable polymer solution on wound healing. (**a**) Macroscopic aspect of wounds under different treatment; (**b**) H&E-stained histological sections of mouse epidermal tissue (3 μm sections). Original magnifications × 400, the photomicrographs are 300 microns on each edge; (**c**) Mixture of photocurable polymer solution-treated BALB/c mice had a significantly higher histological score compared to untreated groups (* *p* < 0.05; *n* = 3).

Wound recoveries by self-recovery or treatment with some drugs usually have obvious differences. Wound area, distribution of subcutaneous cells, and number of hair follicles can provide clues regarding the nature of wound recovery. *S. aureus*, a facultative anaerobic Gram-positive coccal bacterium, is frequently part of skin flora, and it is the most common species of staphylococci to cause staph infections. It also the one of the five most common causes of nosocomial infections. Figure 5 shows the effects of a mixture of photocurable polymer solution on wound healing. The wounds were infected by *S. aureus* on day 1, and then divided into treated and untreated groups. Figure 5a indicates the area of the treated and untreated wounds on different days, which can be referred as the scores for wound recovery. An irregular scar and diffusion of secretions on the wounds of the untreated group on day 13 may not be suitable as a reference for the result of wound repair. The number of neutrophils and fibroblasts alternatively provide a reliable condition of wound recovery without the interference of wound secretions. The percentage of the area of epidermal tissue occupied by neutrophils and fibroblasts will be referred to for wound scores. As shown in Figure 5b, the wound scores were evaluated in a representative wound from the H&E-stained histological sections of mouse epidermal tissue (400×, 3 μm of each edge). Figure 5c shows the total scores of wound recoveries between treated and untreated groups each in triplicate. The quantitative analysis revealed the treated wound to present higher scores than the untreated group. Concerning wound recovery, the treated wound had more fibroblasts in the early stage on day 3. Furthermore, small lymphocytes were found as early as on day 6, giving extra points to the wound recovery score. In our results, the treated group shows not only that the wound area was quickly reduced, but also that the number of neutrophils decreased, fibroblasts increased, and granulation tissue became thicker. The number of hair follicles was also investigated, as shown in the Supporting Information.

## 4. Conclusions

In this study, we have investigated the effect of photo-sensitization using MB and laser light of a 660 nm wavelength on the key virulence factors of *E. coli*. We also applied GE-BTHE mixed with poly(ethylene glycol) diacrylate (PEGDA, a crosslinker) to increase the speed of wound dressing. The mixture of photocurable polymer solution was found to have fast film-forming, antibacterial, bacteria-killing, and wound-healing properties. Therefore, GE-BTHE can be applied as a good dressing support by UV light-irradiation, which can be combined with antibacterial chitosan and MB as a photodynamic therapy agent. In the future, the GE-BTHE mixture may potentially be used clinically in a wide range of wound infection and healing situations.

## Figures and Tables

**Table 1:** Antibacterial efficacy of differing concentrations of MB, chitosan, and GE-BTHE for *E. coli* and *S. aureus*.

	GE-BTHE		Chitosan		MB (L+)		MB (L−)	
***E. coli***	**Conc.**	**Viable Count**	**Conc.**	**Viable Count**	**Conc.**	**Viable Count**	**Conc.**	**Viable Count**
	**(μg/mL)**	**(10^5^ cfu/mL)**	**(μg/mL)**	**(10^5^ cfu/mL)**	**(μg/mL)**	**(10^5^ cfu/mL)**	**(μg/mL)**	**(10^5^ cfu/mL)**
	800	830	33	870	0.08	2400	0.08	1530
	1600	110	330	11.5	4	13	4	1480
	4000	12.8	3300	1.5	8	11.2	8	130
***S. aureus***	**Conc.**	**Viable Count**	**Conc.**	**Viable Count**	**Conc.**	**Viable Count**	**Conc.**	**Viable Count**
	**(μg/mL)**	**(10^5^ cfu/mL)**	**(μg/mL)**	**(10^5^ cfu/mL)**	**(μg/mL)**	**(10^5^ cfu/mL)**	**(μg/mL)**	**(10^5^ cfu/mL)**
	800	8320	33	8200	0.08	8690	0.08	9180
	1600	7780	330	10	4	6670	4	8890
	4000	5160	3300	3.3	8	6750	8	8420

*E. coli* (control): 1.6 × 10^8^ cfu/mL; *S. aureus* (control): 1.1 × 10^9^ cfu/mL; L+: light; L−: no light.

**Table 2:** Bacteriostasis of Gram-positive and Gram-negative in different GE-BTHE/chitosan ratio.

Bacteria	GE-BTHE/Chitosan Ratio	Bacteriostatic Diameter (mm)
*E. coli*	1/3	13
*E. coli*	3/3	10
*E. coli*	5/3	10
*E. coli*	3/1	9
*S. aureus*	1/3	14
*S. aureus*	3/3	15
*S. aureus*	3/1	10

## References

[1] Simoes D., Miguel S.P., Ribeiro M.P., Coutinho P., Mendonca A.G., Correia I.J. (2018). Recent advances on antimicrobial wound dressing: A review. Eur J. Pharm. Biopharm..

[2] Zeng R., Lin C., Lin Z., Chen H., Lu W., Lin C., Li H. (2018). Approaches to cutaneous wound healing: Basics and future directions. Cell Tissue Res..

[3] Chen S.M., Yang J.H., Hung S.J., Wang C.H., Hsu Y.H. (2017). Melting graft wound syndrome. Dermatol. Sin..

[4] Wardhana A., Djan R., Halim Z. (2017). Bacterial and antimicrobial susceptibility profile and the prevalence of sepsis among burn patients at the burn unit of cipto mangunkusumo hospital. Ann. Burns Fire Disasters.

[5] Tang H., Zhang P., Kieft T.L., Ryan S.J., Baker S.M., Wiesmann W.P., Rogelj S. (2010). Antibacterial action of a novel functionalized chitosan-arginine against gram-negative bacteria. Acta Biomater..

[6] Dong Y., Li X., Tian L., Bell T., Sammons R.L., Dong H. (2011). Towards long-lasting antibacterial stainless steel surfaces by combining double glow plasma silvering with active screen plasma nitriding. Acta Biomater..

[7] Bastos M.d.C.d.F., Coutinho B.G., Coelho M.L.V. (2010). Lysostaphin: A staphylococcal bacteriolysin with potential clinical applications. Pharmaceuticals.

[8] Dijkmans A.C., Zacarías N.V.O., Burggraaf J., Mouton J.W., Wilms E., van Nieuwkoop C., Touw D.J., Stevens J., Kamerling I.M.C. (2017). Fosfomycin: Pharmacological, clinical and future perspectives. Antibiotics.

[9] Lu S., Gao W., Gu H.Y. (2008). Construction, application and biosafety of silver nanocrystalline chitosan wound dressing. Burns.

[10] Michel H., Hermans M. (2007). Silver-containing dressings and the need for evidence. Adv. Skin Wound Care J. Prev. Heal..

[11] Leaper D. (2012). International consensus: Appropriate use of silver dressings in wounds. Int. Wound J..

[12] Chen X., Schluesener H.J. (2008). Nanosilver: A nanoproduct in medical application. Toxicol. Lett..

[13] Yuan Y.-G., Peng Q.-L., Gurunathan S. (2017). Effects of silver nanoparticles on multiple drug-resistant strains of staphylococcus aureus and pseudomonas aeruginosa from mastitis-infected goats: An alternative approach for antimicrobial therapy. Int. J. Mol. Sci..

[14] Mohammed A., Al-Qahtani A., Al-Mutairi A., Al-Shamri B., Aabed K. (2018). Antibacterial and cytotoxic potential of biosynthesized silver nanoparticles by some plant extracts. Nanomaterials.

[15] Hsin Y.H., Chen C.F., Huang S., Chih T.S., Lai P.S., Chueh P.J. (2008). The apoptotic effect of nanosilver is mediated by a ros- and jnk-dependent mechanism involving the mitochondrial pathway in nih3t3 cells. Toxicol. Lett..

[16] Grace J.L., Huang J.X., Cheah S.-E., Truong N.P., Cooper M.A., Li J., Davis T.P., Quinn J.F., Velkov T., Whittaker M.R. (2016). Antibacterial low molecular weight cationic polymers: Dissecting the contribution of hydrophobicity, chain length and charge to activity. RSC Adv..

[17] Grace J.L., Elliott A.G., Huang J.X., Schneider E.K., Truong N.P., Cooper M.A., Li J., Davis T.P., Quinn J.F., Velkov T. (2017). Cationic acrylate oligomers comprising amino acid mimic moieties demonstrate improved antibacterial killing efficiency. J. Mater. Chem. B.

[18] Paluck S.J., Maynard H.D. (2017). Structure activity relationship of heparin mimicking polymer p(ss-co-pegma): Effect of sulfonation and polymer size on fgf2-receptor binding. Polym. Chem..

[19] Weintraub S., Shpigel T., Harris L.G., Schuster R., Lewis E.C., Lewitus D.Y. (2017). Astaxanthin-based polymers as new antimicrobial compounds. Polym. Chem..

[20] Mei L., Fan R., Li X., Wang Y., Han B., Gu Y., Zhou L., Zheng Y., Tong A., Guo G. (2017). Nanofibers for improving the wound repair process: The combination of a grafted chitosan and an antioxidant agent. Polym. Chem..

[21] Zhao J., Xu R., Luo G., Wu J., Xia H. (2016). Self-healing poly(siloxane-urethane) elastomers with remoldability, shape memory and biocompatibility. Polym. Chem..

[22] Benhabiles M.S., Salah R., Lounici H., Drouiche N., Goosen M.F.A., Mameri N. (2012). Antibacterial activity of chitin, chitosan and its oligomers prepared from shrimp shell waste. Food Hydrocolloids.

[23] Goy R.C., Morais S.T.B., Assis O.B.G. (2016). Evaluation of the antimicrobial activity of chitosan and its quaternized derivative on e. Coli and s. Aureus growth. Rev. Bras. Farmacogn..

[24] Kong M., Chen X.G., Xing K., Park H.J. (2010). Antimicrobial properties of chitosan and mode of action: A state of the art review. Int. J. Food Microbiol..

[25] Goy R.C., Britto D.d., Assis O.B.G. (2009). A review of the antimicrobial activity of chitosan. Polímeros.

[26] Jarmila V., Eva V. (2011). Chitosan derivatives with antimicrobial, antitumour and antioxidant activities–a review. Curr. Pharm. Des..

[27] No H.K., Young Park N., Ho Lee S., Meyers S.P. (2002). Antibacterial activity of chitosans and chitosan oligomers with different molecular weights. Int. J. Food Microbiol.

[28] Li J., Wu Y., Zhao L. (2016). Antibacterial activity and mechanism of chitosan with ultra high molecular weight. Carbohydr. Polym..

[29] Tin S., Sakharkar K.R., Lim C.S., Sakharkar M.K. (2009). Activity of chitosans in combination with antibiotics in pseudomonas aeruginosa. Int. J. Biol Sci.

[30] Wong T.W., Wu E.C., Ko W.C., Lee C.C., Hor L.I., Huang I.H. (2017). Photodynamic inactivation of methicillin-resistant staphylococcus aureus by indocyanine green and near infrared light. Dermatol. Sin..

[31] Caffarel-Salvador E., Kearney M.-C., Mairs R., Gallo L., Stewart S., Brady A., Donnelly R. (2015). Methylene blue-loaded dissolving microneedles: Potential use in photodynamic antimicrobial chemotherapy of infected wounds. Pharmaceutics.

[32] Van Straten D., Mashayekhi V., de Bruijn H., Oliveira S., Robinson D. (2017). Oncologic photodynamic therapy: Basic principles, current clinical status and future directions. Cancers.

[33] Lai P.S., Lou P.J., Peng C.L., Pai C.L., Yen W.N., Huang M.Y., Young T.H., Shieh M.J. (2007). Doxorubicin delivery by polyamidoamine dendrimer conjugation and photochemical internalization for cancer therapy. J. Control. Release.

[34] Shieh M.J., Peng C.L., Lou P.J., Chiu C.H., Tsai T.Y., Hsu C.Y., Yeh C.Y., Lai P.S. (2008). Non-toxic photo-triggered gene transfection by pamam-porphyrin conjugates. J. Control. Release.

[35] Peng C.L., Lai P.S., Lin F.H., Wu S.Y.H., Shieh M.J. (2009). Dual chemotherapy and photodynamic therapy in an HT-29 human colon cancer xenograft model using SN-38-loaded chlorin-core star block copolymer micelles. Biomaterials.

[36] Lai S.-M., Chiou Y.-C., Chen G.-F., Liao M.-Y., Tzen J.T.C., Lai P.S. (2016). Enhanced nuclear localization of photosensitizer using artificial oil bodies for photodynamic therapy. Smart Sci..

[37] Lin R.-K., Chiu C.-I., Hsu C.-H., Lai Y.-J., Venkatesan P., Huang P.-H., Lai P.-S., Lin C.-C. (2018). Photocytotoxic copper(ii) complexes with schiff-base scaffolds for photodynamic therapy. Chem. Eur. J..

[38] Tavares A., Carvalho C.M.B., Faustino M.A., Neves M.G.P.M.S., Tomé J.P.C., Tomé A.C., Cavaleiro J.A.S., Cunha Â., Gomes N.C.M., Alves E. (2010). Antimicrobial photodynamic therapy: Study of bacterial recovery viability and potential development of resistance after treatment. Mar. Drugs.

[39] Ruiz-González R., Agut M., Reddi E., Nonell S. (2015). A comparative study on two cationic porphycenes: Photophysical and antimicrobial photoinactivation evaluation. Int. J. Mol. Sci..

[40] Dai T., Huang Y.-Y., Hamblin M.R. (2009). Photodynamic therapy for localized infections—State of the art. Photodiagn. Photodyn. Ther..

[41] Fila G., Kasimova K., Arenas Y., Nakonieczna J., Grinholc M., Bielawski K.P., Lilge L. (2016). Murine model imitating chronic wound infections for evaluation of antimicrobial photodynamic therapy efficacy. Front. Microbiol..

[42] Patrulea V., Ostafe V., Borchard G., Jordan O. (2015). Chitosan as a starting material for wound healing applications. Eur J. Pharm. Biopharm..

[43] Ding F.-C., Hsu S.-h., Chiang W.-Y. (2008). Synthesis of a new photoreactive gelatin with btda and hema derivatives. J. Appl. Polym. Sci..

[44] Mazzoccoli J.P., Feke D.L., Baskaran H., Pintauro P.N. (2010). Mechanical and cell viability properties of crosslinked low- and high-molecular weight poly(ethylene glycol) diacrylate blends. J. Biomed. Mater. Res. A.

[45] Altavilla D., Saitta A., Cucinotta D., Galeano M., Deodato B., Colonna M., Torre V., Russo G., Sardella A., Urna G. (2001). Inhibition of lipid peroxidation restores impaired vascular endothelial growth factor expression and stimulates wound healing and angiogenesis in the genetically diabetic mouse. Diabetes.

[46] Shackelford C., Long G., Wolf J., Okerberg C., Herbert R. (2002). Qualitative and quantitative analysis of nonneoplastic lesions in toxicology studies. Toxicol. Pathol..

[47] Tardivo J.P., Del Giglio A., de Oliveira C.S., Gabrielli D.S., Junqueira H.C., Tada D.B., Severino D., de Fátima Turchiello R., Baptista M.S. (2005). Methylene blue in photodynamic therapy: From basic mechanisms to clinical applications. Photodiagn. Photodyn. Ther..

[48] Usacheva M.N., Teichert M.C., Biel M.A. (2001). Comparison of the methylene blue and toluidine blue photobactericidal efficacy against gram-positive and gram-negative microorganisms. Lasers Surg. Med..

[49] Lim E.J., Oak C.H., Heo J., Kim Y.H. (2013). Methylene blue-mediated photodynamic therapy enhances apoptosis in lung cancer cells. Oncol. Rep..

[50] Chen Y., Zheng W., Li Y., Zhong J., Ji J., Shen P. (2008). Apoptosis induced by methylene-blue-mediated photodynamic therapy in melanomas and the involvement of mitochondrial dysfunction revealed by proteomics. Cancer Sci..

[51] Su H.-L., Chou C.-C., Hung D.-J., Lin S.-H., Pao I.C., Lin J.-H., Huang F.-L., Dong R.-X., Lin J.-J. (2009). The disruption of bacterial membrane integrity through ros generation induced by nanohybrids of silver and clay. Biomaterials.

[52] Park J.-H., Moon Y.-H., Bang I.-S., Kim Y.-C., Kim S.-A., Ahn S.-G., Yoon J.-H. (2010). Antimicrobial effect of photodynamic therapy using a highly pure chlorin e6. Lasers Med. Sci..

[53] Caminos D.A., Spesia M.B., Durantini E.N. (2006). Photodynamic inactivation of escherichia coli by novel meso-substituted porphyrins by 4-(3-n,n,n-trimethylammoniumpropoxy)phenyl and 4-(trifluoromethyl)phenyl groups. Photochem. Photobiol. Sci..

[54] Sang L.-Y., Zhou X.-H., Yun F., Zhang G.-L. (2010). Enzymatic synthesis of chitosan–gelatin antimicrobial copolymer and its characterisation. J. Sci. Food Agric..

[55] Vinsova J., Vavrikova E. (2008). Recent advances in drugs and prodrugs design of chitosan. Curr. Pharm. Des..

[56] Zheng L.-Y., Zhu J.-F. (2003). Study on antimicrobial activity of chitosan with different molecular weights. Carbohydr. Polym..

[57] Bellich B., D’Agostino I., Semeraro S., Gamini A., Cesàro A. (2016). “The good, the bad and the ugly” of chitosans. Mar. Drugs.

[58] Rattanaruengsrikul V., Pimpha N., Supaphol P. (2009). Development of gelatin hydrogel pads as antibacterial wound dressings. Macromol. Biosci..

[59] Dale P.D., Sherratt J.A., Maini P.K. (1996). A mathematical model for collagen fibre formation during foetal and adult dermal wound healing. Proc. R. Soc. B.

[60] Xue M., Jackson C.J. (2015). Extracellular matrix reorganization during wound healing and its impact on abnormal scarring. Adv. Wound Care.

[61] Dobić S.N., Jovašević J.S., Vojisavljević M.D., Tomić S.L. (2011). Hemocompatibility and swelling studies of poly(2-hydroxyethyl methacrylate-co-itaconic acid-co-poly(ethylene glycol) dimethacrylate) hydrogels. Hem. Ind..

[62] Wang H.-M., Chou Y.-T., Wen Z.-H., Wang Z.-R., Chen C.-H., Ho M.-L. (2013). Novel biodegradable porous scaffold applied to skin regeneration. PLOS ONE.

[63] Browning M.B., Cereceres S.N., Luong P.T., Cosgriff-Hernandez E.M. (2014). Determination of the in vivo degradation mechanism of pegda hydrogels. J. Biomed. Mater. Res. A.

[64] Yang C., Xu L., Zhou Y., Zhang X., Huang X., Wang M., Han Y., Zhai M., Wei S., Li J. (2010). A green fabrication approach of gelatin/cm-chitosan hybrid hydrogel for wound healing. Carbohydr. Polym..

[65] Li C., Fu R., Yu C., Li Z., Guan H., Hu D., Zhao D., Lu L. (2013). Silver nanoparticle/chitosan oligosaccharide/poly(vinyl alcohol) nanofibers as wound dressings: A preclinical study. Int. J. Nanomed..

[66] Peng C.-C., Yang M.-H., Chiu W.-T., Chiu C.-H., Yang C.-S., Chen Y.-W., Chen K.-C., Peng R.Y. (2008). Composite nano-titanium oxide–chitosan artificial skin exhibits strong wound-healing effect—An approach with anti-inflammatory and bactericidal kinetics. Macromol. Biosci..

[67] Mao J.S., Zhao L.G., Yin Y.J., Yao K.D. (2003). Structure and properties of bilayer chitosan-gelatin scaffolds. Biomaterials.

